# Evaluation of low-cost techniques to detect sickle cell disease and β-thalassemia: an open-label, international, multicentre study

**DOI:** 10.1016/j.lansea.2025.100571

**Published:** 2025-03-29

**Authors:** Pranav Shrestha, Hendrik Lohse, Christopher Bhatla, Heather McCartney, Alaa Alzaki, Navdeep Sandhu, Pardip Kumar Oli, Sanjeev Chaudhary, Ali Amid, Rodrigo Onell, Nicholas Au, Hayley Merkeley, Videsh Kapoor, Rajan Pande, Boris Stoeber

**Affiliations:** a Department of Mechanical Engineering, The University of British Columbia, 2054-6250 Applied Science Lane, Vancouver, British Columbia, V6T 1Z4, Canada; b Department of Mathematics and Computer Science, Eindhoven University of Technology, Groene Loper 3, 5612 AE, Eindhoven, the Netherlands; c Faculty of Medicine, The University of British Columbia, 317-2194 Health Sciences Mall, Vancouver, British Columbia, V6T 1Z3, Canada; d Division of Hematology & Oncology, BC Children's Hospital, 4480 Oak Street, Vancouver, British Columbia, V6H 3V4, Canada; e John Hopkins Aramco Healthcare, Dahran, Eastern Province, Saudi Arabia; f Adult Red Cell Disorders Program of BC and Yukon, St. Paul's Hospital, 1081 Burrard Street, Vancouver, British Columbia, V6Z 1Y6, Canada; g Mount Sagarmatha Polyclinic and Diagnostic Center, Nepalgunj, Bheri Zone, Province No-5, Nepal; h Department of Pathology, Bheri Hospital, Nepalgunj, Bheri Zone, Province No-5, Nepal; i Department of Internal Medicine, Bheri Hospital, Nepalgunj, Bheri Zone, Province No-5, Nepal; j Department of Electrical and Computer Engineering, The University of British Columbia, 2332 Main Mall, Vancouver, British Columbia, V6T 1Z4, Canada

**Keywords:** Sickle cell disease, Haemoglobinopathies, Thalassemia

## Abstract

**Background:**

Sickle cell disease (SCD) persists as a major global health problem, disproportionately affecting children in low- and middle-income countries (LMIC). Accurate and low-cost point-of-care techniques are urgently needed in LMIC to detect carrier or disease forms with haemoglobin S (HbS) and other variants like β-thalassemia.

**Methods:**

An open-label, international, multicentre study was conducted at clinical sites in Nepal and Canada. Blood samples were collected from healthy volunteers (HbAA) and participants with known haemoglobinopathies (HbA/β-thalassemia, HbAS, HbS/β-thalassemia, HbSS). The performance of six low-cost tests (Conventional sickling test; HbS solubility test; HemoTypeSC; Sickle SCAN; Gazelle Hb variant test; Automated sickling test using automated microscopy and machine learning) was evaluated against HPLC (ClinicalTrials.gov Identifier: NCT05506358).

**Findings:**

Between September 2022 and March 2023, we enrolled 138 participants (aged 2–74 years; 59% female, 41% male) at clinical sites in Nepal and Canada. Four low-cost tests (HemoTypeSC, Sickle SCAN, Gazelle, and automated sickling), which could identify phenotypes, detected severe SCD (HbSS, HbS/β-thalassemia) accurately (sensitivity >96%; specificity >99%). In contrast, for carrier forms, HemotypeSC and Sickle SCAN only detected HbAS (sensitivity >97%; specificity 100%) and not HbA/β-thalassemia (sensitivity 0%; specificity 100%), while Gazelle detected HbAS (sensitivity 100%, specificity 100%) and HbA/β-thalassemia (sensitivity 91%, specificity 99%), and automated sickling test detected both trait conditions (HbAS and HbA/β-thalassemia; sensitivity 85%, specificity 85%).

**Interpretation:**

When HbS co-exists with β-thalassemia, Gazelle and automated sickling test accurately identify severe SCD and carrier forms. However, HemotypeSC and Sickle SCAN miss β-thalassemia trait, and need to be complemented with other low-cost tests.

**Funding:**

10.13039/501100005247UBC10.13039/501100000241PSI, 10.13039/501100001804Canada Research Chairs, 10.13039/501100005247UBC HIFI Awards, 10.13039/501100005247UBC 4YF, Naiman Vickars Endowment fund.

**Research in context**

**Evidence before this study**

There is clear evidence that the highest burden for sickle cell disease (SCD) exists in low- and middle-income countries (LMICs). Despite massive advancements in treatment and care, including groundbreaking results and first regulatory approvals for gene-therapy based treatment for patients with SCD or transfusion-dependent β-thalassemia, a vast majority of affected communities lack access to diagnosis (including newborn screening) and basic treatment. We searched PubMed between August 2020 and June 2023 for articles and studies published within the last 20 years, using search terms such as “sickle cell disease”, “sickle cell trait”, “beta-thalassemia”, “HemotypeSC”, “Sickle SCAN”, “Gazelle” OR “Hemechip”, AND “low-cost” OR “point-of-care”. Several promising techniques for point-of-care detection of SCD in LMIC have been developed and commercialised (notably, HemotypeSC, Sickle SCAN, and Gazelle), and many clinical studies have validated their high sensitivity and specificity to detect haemoglobin S, even in field settings. However, the limitations of implementing these tools in the presence of other related mutations, especially β-thalassemia, have not been highlighted or explored sufficiently. Furthermore, clinical studies lack simultaneous implementation and comparison of more than two of these low-cost alternatives in LMIC. In this study, suitable point-of-care diagnostic candidates were identified based on current clinical practices and locally available tests in Nepal and Canada, conventional screening techniques described in major haematology books, and reports or reviews on recently commercialised tests.

**Added value of this study**

To our knowledge, this is the first clinical study to concurrently test six different low-cost alternatives to expensive laboratory-based methods for detecting SCD and β-thalassemia. The study was designed in collaboration with a multi-disciplinary and international team to evaluate the feasibility of implementing the low-cost techniques in low-resource settings. We provide guidelines (specific to region, resource, and haemoglobin variant prevalence) for selecting low-cost techniques and discuss pros and cons of the different options. Additionally, we introduce results for a new technique based on automated microscopy of the sickling test and machine-learning based classification.

**Implications of all the available evidence**

One of the first steps to ensuring equitable access to SCD treatment and care, which is urgently needed to reduce the disease mortality or morbidity, is to effectively screen communities using accurate, low-cost point-of-care techniques. In addition to providing performance metrics for different low-cost techniques, we provide recommendations, guidelines and limitations related to each technique. The evidence provided here can equip health workers, researchers, policy makers, organizations and governments with a more complete picture and more relevant information than before regarding suitable low-cost techniques that can be deployed in their local communities, especially if additional mutations such as β-thalassemia are prevalent.

## Introduction

Sickle haemoglobin or haemoglobin S (HbS) results from a single point mutation (β6^Glu→Val^) in the β-globin gene^1^^,^^2^ and presents an increasing global health problem, particularly in resource limited environments.^3^^,^^4^ The prevalence of sickle cell disease (SCD) increased by 41.4% from 5.46 million cases in 2000 to 7.74 million cases in 2021, with the highest mortality burden in children.^5^ The most common and severe form of SCD is sickle cell anaemia (SCA), which results from homozygous inheritance of HbS. Other compound heterozygous forms of SCD exist (most notably with the β-globin variants, haemoglobin C, and with β-thalassemia) and have variable phenotypes.^1^^,^^4^^,^^6^ SCD is a multi-system disorder affecting nearly every organ in the body.^4^ Polymerisation of deoxygenated HbS distorts red blood cells (RBCs) into characteristic sickle shaped cells^2^ promoting chronic vaso-occlusion, haemolysis, inflammation, and progressive organ damage.^1^^,^^6^ Morbidity and mortality improve with early diagnosis, infection prevention, and disease modifying therapy including RBC transfusions and hydroxyurea.^1^^,^^4^ On the other hand, the heterozygous form of HbS known as sickle cell trait (SCT) has an estimated annual prevalence and birth rate of 300 million^7^ and 5.48 million,^1^ respectively. SCT is typically not associated with haematological abnormalities,^2^ thus may go undetected without effective screening programmes.

One of the first steps to addressing SCD in low- and middle-income countries (LMIC) is to break the diagnostic barrier. Low-cost and rapid point-of-care diagnostic devices are required for early detection. The current laboratory techniques are expensive, inaccessible and personnel dependent, which prevents timely and equitable diagnosis.^8^ Traditionally, the sickling test and the solubility test have been used as low-cost screening tools for detecting the presence of HbS, but neither can distinguish between SCT and SCD.^2^^,^^9^ Recently, a few promising low-cost point-of-care techniques, such as two lateral flow-assays (HemoTypeSC, Silver Lake Research, USA, and Sickle SCAN, BioMedomics Inc., USA) and a portable electrophoresis instrument (Gazelle Diagnostic Device, Hemex Health, USA), have been commercialised and tested in several countries with reported sensitivity and specificity >92% to detect SCD.^10^, ^11^, ^12^, ^13^, ^14^, ^15^, ^16^, ^17^ The lateral flow assays, HemoTypeSC and Sickle SCAN, can identify phenotypes containing haemoglobin A (HbA, *i.e.* the most common form of adult haemoglobin), HbS and haemoglobin C (HbC), but not β-thalassemia. The Gazelle Hb variant test can identify phenotypes containing HbA, HbS, HbC, and other variants, including β-thalassemia.^18^ Other point-of-care diagnostic techniques^19^^,^^20^ have been developed by research groups as well, but the techniques discussed above include conventionally used or commercially available tests.

When screening for HbS in communities, it is also essential to identify other commonly prevalent haemoglobin variants, such as β-thalassemia, prevalent in the Middle East, Mediterranean countries and South Asia,^1^^,^^6^^,^^21^ or HbC, prevalent in West Africa.^3^ Such identifications help prevent genetic transmissions resulting in severe forms of SCD (compound heterozygous or homozygous HbS). After screening, individuals identified with disease states can be provided with the appropriate treatment or medical attention, while individuals with trait conditions can be provided with genetic counselling to understand the risks of passing variant genes to children.

In this study, we evaluated six low-cost tests against the local gold standard, haemoglobin high performance liquid chromatography (Hb HPLC). The low-cost tests included two conventional tests (conventional sickling test, solubility test), three recently commercialized techniques (HemoTypeSC, Sickle SCAN, Gazelle Hb variant test), and one newly developed technique (automated sickling test^22^^,^^23^). The purpose of this study was to determine the accuracy of the low-cost point-of-care techniques for screening and detecting SCD, SCT, and β-thalassemia, and to evaluate their feasibility to supplement or replace gold standard tests. Here, we report the first combined comparison of several low-cost techniques, using identical blood samples for all tests (including Hb HPLC) from each participant, to detect HbS and β-thalassemia in participants from Nepal and Canada.

## Methods

### Study design and participants

An open-label, international, multicentre study was conducted at (i) St. Paul's Hospital and BC Children's Hospital, two hospitals in Vancouver, Canada, and (ii) Mount Sagarmatha Polyclinic and Diagnostic Center, a clinical site in Nepalgunj, Nepal from September 2022 to March 2023. Individuals with SCD (HbSS and HbS/β-thalassemia), SCT (HbAS), and β-thalassemia trait (HbA/β-thalassemia), and individuals without known haemoglobinopathies (HbAA) were recruited for the study, with participants forming a consecutive series. Participants were excluded for pregnancy, blood transfusion within the last three months, and presence of HbC (due to low prevalence in Nepal). Use of hydroxyurea was not an exclusion criterion. All participants except normal controls had been diagnosed prior to the study with Hb HPLC. During the study, Hb HPLC was performed on all participant samples to diagnose haemoglobinopathies, and normal controls had no known haemoglobinopathies based on Hb HPLC (although other factors such as iron deficiency were not measured). Confirmatory β-globin genotyping was not performed for most participants. Participants or their parents or guardians provided informed consents, and the study was approved by institutional/national research ethics boards in Nepal and Canada (ClinicalTrials.gov Identifier: NCT05506358). Details of blood sample collection in Supplementary Information Section 1.

### Conventional and automated sickling tests

The sample preparation of the sickling test^2^^,^^9^ was optimised for high-throughput imaging using a low-cost automated microscope, Octopi.^24^ Images of RBCs were captured 2 h after sample preparation, to enable reagent-induced sickling, and they were evaluated by eye (conventional sickling test) or using machine learning-based algorithms (automated sickling test) (Supplementary Information Section 2.5).

### HbS solubility test

The haemoglobin solubility (or sickle solubility or HbS solubility) test is a common low-cost technique to detect the presence of HbS, without distinguishing between SCT and SCD.^2^ HbS solubility test kits are usually available locally in regions with high HbS prevalence, and two such test kits were used—Sickle-Heme (Great Lakes Diagnostics, Michigan, USA) in Canada and Sicklevue (Microxpress, Goa, India) in Nepal. Both kits were used according to the company instructions (Supplementary Information Section 2.1).

### Lateral flow assays

HemoTypeSC^11^^,^^12^ is a competitive binding-based lateral flow assay, and Sickle SCAN^14^ is a non-competitive binding-based (or sandwich) lateral flow immunoassay. Both assays detect HbA, HbS and HbC, while differentiating between normal, carrier and disease forms. The testing procedure for both lateral flow assays followed the company's instructions for use (Supplementary Information Sections 2.2 and 2.3).

### Gazelle Hb variant test

The Gazelle Hb variant test is a portable electrophoresis machine that can identify haemoglobin disorders including sickle cell disease and β-thalassemia.^17^^,^^18^ The testing procedure (Supplementary Information Section 2.4) has many more steps than the lateral flow assays (HemoTypeSC and Sickle SCAN), but unlike the lateral flow assays, the Gazelle can also detect β-thalassemia.^18^

### Hb HPLC

Hb HPLC is one of the common modalities used clinically for diagnosing haemoglobinopathies.^2^ At the respective clinical sites in Nepal and Canada, trained laboratory technologists performed Hb HPLC with the blood samples on the D10 Haemoglobin Testing System (Bio-Rad Laboratories Inc., California, USA) in Nepal, or on the BioRad Variant II (Bio-Rad Laboratories Inc., California, USA) in Canada, following the manufacturer's guidelines. A maximum of 10 samples were automatically tested sequentially in one run on either of the systems, even though the BioRad Variant II allowed loading up to 50 samples at a time. The results of the HPLC were interpreted by a medical laboratory technologist in Nepal and a hematopathologist in Canada to provide the reference diagnosis for each participant. Hb HPLC was the reference test for all the low-cost tests (index tests), and HPLC results were taken as the true phenotype for data analysis.

### Data analysis

The sensitivity, specificity, positive predictive value (PPV) and negative predictive value (NPV) of the index tests or low-cost techniques were evaluated using true positive (TP), true negative (TN), false positive (FP), and false negative (FN) results, which were evaluated against Hb HPLC as the reference test (details and equations in Supplementary Information Section 3).

### Role of the funding source

The funders had no role in conduct of the study, interpretation, writing the manuscript or decision to submit. No authors were paid to write this article by any company, organisation or agency.

## Results

### Study participants

**Fig. 1:**
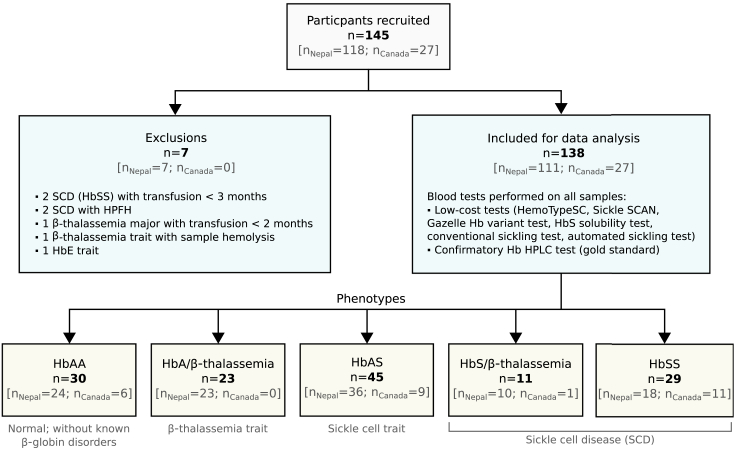
Study flow diagram and participant numbers. A total of 145 participants were recruited in Nepal (118) and Canada (27). After confirmatory Hb HPLC diagnoses, 7 samples were excluded and a total of 138 participant samples were tested with 6 low-cost tests and Hb HPLC. n, number of participants or samples; n_Nepal_, number in Nepal; n_Canada_, number in Canada; SCD, sickle cell disease; HPFH, hereditary persistence of foetal haemoglobin; Hb, haemoglobin; HPLC, high-performance liquid chromatography.

A total of 145 participants (Fig. 1 and Table 1) were recruited including 118 in Nepal between November 2022 to December 2022 and 27 in Canada between September 2022 and March 2023. Out of these, seven samples were excluded due to either (i) blood transfusion within the past three months (for two SCD samples and one β-thalassemia major sample), (ii) presence of a haemoglobinopathy not considered in the study, such as haemoglobin E (HbE) in one sample, and hereditary persistence of foetal haemoglobin (HPFH) in two samples, or (iii) defect in sample (haemolysis observed in microscopic images for one sample). The final data analysis included results from 138 participants for comparison of low-cost tests with Hb HPLC. In Nepal and Canada, the samples were from 30 (21.7%) unaffected (HbAA), 23 (16.7%) β-thalassemia trait (HbA/β-thalassemia), 45 (32.6%) sickle cell trait (HbAS), and 40 (29.0%) sickle cell disease (11 HbS/β-thalassemia and 29 HbSS) participants, all of whose diagnoses were confirmed with Hb HPLC.

**Table 1:** Demographics for population included in the study, including the number of participants and percentage in parenthesis.

	Sex		Age		
	Female	Male	2–18 years	19–49 years	≥50 years
Nepal	67 (60%)	44 (40%)	30 (27%)	72 (65%)	9 (8%)
Canada	14 (52%)	13 (48%)	3 (11%)	22 (81%)	2 (7%)
Total	81 (59%)	57 (41%)	33 (24%)	94 (68%)	11 (8%)

In Canada, molecular confirmation of the genotype was used to distinguish between cases of HbSS (11 cases) and HbS/β-thalassemia (1 case) if mean corpuscular volume (MCV) was decreased with increased HbA_2_ levels in Hb HPLC results. In Nepal, a cut-off value of 4% HbA_2_ in Hb HPLC results was used to identify β-thalassemia, following the guidelines: (i) HbA_2_ <3.5%, β-thalassemia absent; (ii) 3.6% ≤HbA_2_ ≤ 4.0%, borderline case (recommended for molecular testing of genotype); (iii) HbA_2_ >4.0%, β-thalassemia present (presence of HbS peak differentiated HbS/β-thalassemia from HbA/β-thalassemia). In our study population, all participants with HbA/β-thalassemia had HbA_2_ ≥4.9% and all participants with HbS/β-thalassemia had HbA_2_ ≥4.1%. The summary of HPLC results (mean and range) for the major peaks from Hb HPLC (HbA, HbF, HbA_2_, HbS) for all participants in Nepal and Canada are listed in Supplementary Table S1 in the Supplementary Information Section 4.

Participants’ age ranged from 2 to 74 years (24% 2–18 years, 68% 19–49 years, and 8% 50 years and over), with 59% female and 41% male (Table 1). Hydroxyurea was used by 17/18 (94%) of HbSS participants and 7/10 (70%) of HbS/β-thalassemia participants in Nepal, and by 10/11 (91%) of HbSS participants and 1/1 (100%) of HbS/β-thalassemia participants in Canada. No adverse events were observed.

### Analysis by presence of haemoglobin variant

Table 2 summarises the ability of the different low-cost tests to detect the presence of haemoglobin variants in samples from 138 participants in Nepal and Canada: HbA for normal (HbAA), β-thalassemia trait (HbA/β-thalassemia) or SCT participants (HbAS); HbS for SCT (HbAS) or SCD participants (HbSS and HbS/β-thalassemia); and β-thalassemia for β-thalassemia trait (HbA/β-thalassemia) or compound heterozygous SCD (HbS/β-thalassemia). The sensitivity to detect the presence of HbS mutation was high (>98%) for all low-cost tests, including the solubility test and the conventional sickling test, which provided a positive test result in the presence of HbS (*i.e.* for HbAS, HbSS, and HbS/β-thalassemia), while not differentiating between SCT and SCD. The sensitivity to detect HbA was high (≥99%) for the Gazelle, HemoTypeSC and Sickle SCAN, and was lower (54–55%) for the solubility test and the conventional sickling test because of their inability to identify HbA from SCT cases. The overall or balanced sensitivity for detecting HbA and HbS (Ov2 in Table 2) was >99% for the Gazelle and the lateral flow assays (HemoTypeSC and Sickle SCAN), and around 77% for the solubility test and the conventional sickling test.

**Table 2:** Sensitivity, specificity, positive predictive value (PPV) and negative predictive value (NPV) for 5 low-cost tests to detect different haemoglobin variants in samples from 138 participants in Nepal and Canada, i.e. detection of the presence of HbA in HbAA, HbA/β-thalassemia or HbAS; presence of HbS in HbS/β-thalassemia or HbSS; and presence of β in HbA/β-thalassemia or HbS/β-thalassemia.

Test^a^	Hb	Sensitivity		Specificity		PPV^d^		NPV	
		TP/(TP + FN)	% (95% CI)	TN/(TN + FP)	% (95% CI)	TP/(TP + FP)	% (95% CI)	TN/(TN + FN)	% (95% CI)
Gazelle	A	98/98	100 (96–100)	40/40	100 (91–100)	98/98	100 (96–100)	40/40	100 (91–100)
	S	85/85	100 (96–100)	53/53	100 (93–100)	85/85	100 (96–100)	53/53	100 (93–100)
	β	21/34	62 (45–76)	102/104	98 (93–99)	21/23	91 (73–98)	102/115	89 (82–93)
	**Ov**^b^		**87 (80–92)**		**99 (95–100)**		**97 (92–99)**		**96 (91–99)**
	**Ov2**^c^		**100 (96–100)**		**100 (92–100)**		**100 (96–100)**		**100 (92–100)**
HemoTypeSC^e^	A	97/98	99 (94–100)	40/40	100 (91–100)	97/97	100 (96–100)	40/41	98 (87–100)
	S	85/85	100 (96–100)	53/53	100 (93–100)	85/85	100 (96–100)	53/53	100 (93–100)
	β	0/34	0 (0–10)	104/104	100 (96–100)	0/0	–	104/138	75 (68–82)
	**Ov**^b^		**66 (57–75)**		**100 (96–100)**		**-**		**91 (85–95)**
	**Ov2**^c^		**100 (95–100)**		**100 (92–100)**		**100 (96–100)**		**99 (90–100)**
Sickle SCAN^e^	A	98/98	100 (96–100)	40/40	100 (91–100)	98/98	100 (96–100)	40/40	100 (91–100)
	S	85/85	100 (96–100)	53/53	100 (93–100)	85/85	100 (96–100)	53/53	100 (93–100)
	β	0/34	0 (0–10)	104/104	100 (96–100)	0/0	–	104/138	75 (68–82)
	**Ov**^b^		**67 (57–75)**		**100 (96–100)**		**-**		**92 (85–96)**
	**Ov2**^c^		**100 (96–100)**		**100 (92–100)**		**100 (96–100)**		**100 (92–100)**
Solubility^e^	A	53/98	54 (44–64)	40/40	100 (91–100)	53/53	100 (93–100)	40/85	47 (37–58)
	S	85/85	100 (96–100)	53/53	100 (93–100)	85/85	100 (96–100)	53/53	100 (93–100)
	β	0/34	0 (0–10)	104/104	100 (96–100)	0/0	–	104/138	75 (68–82)
	**Ov**^b^		**51 (42–61)**		**100 (96–100)**		**-**		**74 (66–81)**
	**Ov2**^c^		**77 (68–85)**		**100 (92–100)**		**100 (95–100)**		**74 (62–83)**
Conventional sickling^e^	A	54/98	55 (45–65)	40/40	100 (91–100)	54/54	100 (93–100)	40/84	48 (37–58)
	S	84/85	99 (94–100)	53/53	100 (93–100)	84/84	100 (96–100)	53/54	98 (90–100)
	β	0/34	0 (0–10)	104/104	100 (96–100)	0/0	–	104/138	75 (68–82)
	**Ov**^b^		**51 (42–60)**		**100 (96–100)**		**-**		**74 (66–80)**
	**Ov2**^c^		**77 (67–84)**		**100 (92–100)**		**100 (95–100)**		**73 (61–82)**

Acronyms: PPV, positive predictive value; NPV, negative predictive value; TP, true positive; FN, false negative; TN, true negative; FP, false positive; CI, confidence interval.

Details of calculations in Supplementary Tables S2–S6.

a Low-cost tests or index tests, whose metrics were calculated against reference test Hb HPLC; the automated sickling test is not included in this table since data was trained/tested to detect phenotypes (Table 3) rather than haemoglobin variants.

b Ov: Overall or balanced metrics calculated as averages of metrics for A, S, and β

c Ov2: Overall or balanced metrics calculated as averages of metrics for A and S (excluding β).

d Dashes indicate zero division errors for PPV, with TP + FP = 0.

e These tests are not intended to detect β-thalassemia (hence 0% sensitivity), but are included in the table to highlight the decrease in overall sensitivity to detect A, S, and β (relevant only in regions where HbS and β-thalassemia are both prevalent).

Out of all the low-cost tests in Table 2, only the Gazelle detected the presence of β-thalassemia and had a sensitivity and specificity to detect β-thalassemia (in HbA/β-thalassemia and HbS/β-thalassemia) of 62% and 98%, respectively. Although the Gazelle identified β-thalassemia in β-thalassemia trait participants, it did not definitively identify β-thalassemia from any of the ten HbS/β-thalassemia participants and identified them as HbSS instead while providing a message that β-thalassemia could not be ruled out. The overall or balanced sensitivity for detecting all three variants (Ov in Table 2) was around 87% for the Gazelle, 66–67% for the lateral flow assays, and around 51% for the solubility test and the conventional sickling test (Table 2).

### Analysis by phenotype

Table 3 summarizes the ability of four low-cost tests (excluding solubility and conventional sickling tests) to detect different phenotypes in samples from 138 participants from Nepal and Canada. The sensitivity to detect HbAA, HbAS and HbSS was high (>96%) for the Gazelle and the lateral flow-assays. This is in agreement with previous studies,^10^, ^11^, ^12^, ^13^, ^14^, ^15^, ^16^, ^17^^,^^25^ focused mostly on detection of HbS (or HbC) mutations and not of β-thalassemia. The lateral flow assays were not designed to identify HbA/β-thalassemia and HbS/β-thalassemia, whereas the Gazelle and the automated sickling test identified HbA/β-thalassemia with a sensitivity of 91% and 72%, respectively (Table 3). Additionally, the automated sickling test was able to identify both asymptomatic trait or carrier conditions combined (HbA/β-thalassemia and HbAS) with a higher sensitivity of 85% (Table 3).

**Table 3:** Sensitivity, specificity, positive predictive value (PPV), and negative predictive value (NPV) for 4 low-cost tests to detect different phenotypes in samples from 138 participants in Nepal and Canada.

Test^a^	Hb	Sensitivity		Specificity		PPV^e^		NPV	
		TP/(TP + FN)	% (95% CI)	TN/(TN + FP)	% (95% CI)	TP/(TP + FP)	% (95% CI)	TN/(TN + FN)	% (95% CI)
Gazelle	AA	29/30	97 (83–99)	106/108	98 (93–99)	29/31	94 (79–98)	106/107	99 (95–100)
	Aβ	21/23	91 (73–98)	114/115	99 (95–100)	21/22	96 (78–99)	114/116	98 (94–100)
	AS	45/45	100 (92–100)	93/93	100 (96–100)	45/45	100 (92–100)	93/93	100 (96–100)
	Sβ	0/11	0 (0–26)	126/127	99 (96–100)	0/1	0 (0–79)	126/137	92 (86–95)
	SS	28/29	97 (83–99)	98/109	90 (83–94)	28/39	72 (56–83)	98/99	99 (95–100)
	SCD^b^	40/40	100 (91–100)	98/98	100 (96–100)	40/40	100 (91–100)	98/98	100 (96–100)
	**Ov5**^c^		**77 (59–89)**		**97 (92–99)**		**72 (54–85)**		**98 (93–99)**
	**Ov4**^d^		**97 (85–99)**		**99 (95–100)**		**97 (86–100)**		**99 (95–100)**
HemoTypeSC	AA	30/30	100 (89–100)	85/108	79 (70–85)	30/53	57 (43–69)	85/85	100 (96–100)
	Aβ	0/23	0 (0–14)	115/115	100 (97–100)	0/0	–	115/138	83 (76–89)
	AS	44/45	98 (88–100)	93/93	100 (96–100)	44/44	100 (92–100)	93/94	99 (94–100)
	Sβ	0/11	0 (0–26)	127/127	100 (97–100)	0/0	–	127/138	92 (86–95)
	SS	29/29	100 (88–100)	97/109	89 (82–94)	29/41	71 (55–82)	97/97	100 (96–100)
	SCD^b^	40/40	100 (91–100)	97/98	99 (94–100)	40/41	98 (87–100)	97/97	100 (96–100)
	**Ov5**^c^		**60 (41–76)**		**94 (87–97)**		**-**		**95 (89–98)**
	**Ov4**^d^		**74 (58–86)**		**94 (88–97)**		**-**		**96 (90–98)**
Sickle SCAN	AA	30/30	100 (89–100)	85/108	79 (70–85)	30/53	57 (43–69)	85/85	100 (96–100)
	Aβ	0/23	0 (0–14)	115/115	100 (97–100)	0/0	–	115/138	83 (76–89)
	AS	45/45	100 (92–100)	93/93	100 (96–100)	45/45	100 (92–100)	93/93	100 (96–100)
	Sβ	0/11	0 (0–26)	127/127	100 (97–100)	0/0	–	127/138	92 (86–95)
	SS	29/29	100 (88–100)	98/109	90 (83–94)	29/40	73 (57–84)	98/98	100 (96–100)
	SCD^b^	40/40	100 (91–100)	98/98	100 (96–100)	40/40	100 (91–100)	98/98	100 (96–100)
	**Ov5**^c^		**60 (42–76)**		**94 (88–97)**		**-**		**95 (89–98)**
	**Ov4**^d^		**75 (59–86)**		**95 (89–98)**		**-**		**96 (90–98)**
Automated sickling^f^ (4 groups)	AA	42,800/69,009	62 (50–73)	188,852/207,027	91 (87–94)	42,800/60,975	70 (66–74)	188,852/215,061	88 (83–92)
	Aβ	49,408/69,009	72 (60–81)	179,405/207,027	87 (81–91)	49,408/77,030	64 (61–67)	179,405/199,006	90 (85–94)
	AS	52,024/69,009	75 (64–84)	188,943/207,027	91 (87–94)	52,024/70,108	74 (71–77)	188,943/205,928	92 (87–95)
	SCD^b^	66,786/69,009	97 (90–99)	205,890/207,027	100 (97–100)	66,786/67,923	98 (97–99)	205,890/208,113	99 (96–100)
	**Ov4**^d^		**76 (65–85)**		**92 (88–95)**		**77 (73–80)**		**92 (88–95)**
Automated sickling^f^ (3 groups)	AA	69,177/96,723	72 (62–80)	180,204/193,452	93 (89–96)	69,177/82,425	84 (74–90)	180,204/207,750	87 (82–91)
	Trait^g^	82,109/96,723	85 (76–91)	163,854/193,452	85 (79–89)	82,109/111,707	75 (66–82)	163,854/178,468	92 (87–95)
	SCD^b^	94,115/96,729	97 (92–99)	191,518/193,446	99 (96–100)	94,115/96,043	98 (93–100)	191,518/194,132	99 (96–100)
	**Ov3**^h^		**85 (76–90)**		**92 (88–95)**		**86 (77–91)**		**93 (88–96)**

Acronyms: PPV, positive predictive value; NPV, negative predictive value; TP, true positive; FN, false negative; TN, true negative; FP, false positive; CI, confidence interval.

Details of calculations in Supplementary Tables S7–S14.

a Low-cost tests or index tests, whose metrics were calculated against reference test Hb HPLC; the solubility test and the conventional sickling test only identified Hb variant presence and not phenotypes.

b SCD, Sickle cell disease; including HbS/β-thalassemia and HbSS.

c Ov5: Overall or balanced metrics for 5 groups calculated as averages of metrics for AA, Aβ, AS, Sβ, and SS.

d Ov4: Overall or balanced metrics for 4 groups calculated as averages of metrics for AA, Aβ, AS, and SCD (Sβ + SS).

e Dashes indicate zero division errors for PPV, with TP + FP = 0.

f Result for coverslip-wise morphology-based classification using subspace discriminant classifier for the testing dataset only; aggregated or merged result for 1000 randomized participant-wise splits of training and testing data (80:20 splits) using the same population as the other tests, *i.e*. 138 participants from Nepal and Canada.

g Asymptomatic trait or carrier conditions, including HbA/β-thalassemia and HbAS.

h Ov3: Overall or balanced metrics for 3 groups calculated as averages of metrics for AA, trait (Aβ + AS), and SCD (Sβ + SS).

The Gazelle and the lateral flow-assays misidentified all HbS/β-thalassemia as HbSS (confusion matrices in Supplementary Tables S7–S9), and the instructions for use (for lateral flow assays) or results (for Gazelle) reported that such misclassifications were to be expected. Moreover, the Gazelle also misidentified one HbSS sample as HbS/β-thalassemia (Supplementary Table S7). Since HbS/β-thalassemia and HbSS have similar clinical outcomes,^1^^,^^6^ the ability of the low-cost tests to detect them together (grouped as SCD) is also reported in Table 3. All the four tests in Table 3 (Gazelle, lateral flow assays and automated sickling test) detected SCD with a sensitivity of over 96%, suggesting the feasibility of using any of these low-cost tests to accurately screen for severe disease cases (*i.e.* HbS/β-thalassemia and HbSS). The overall or balanced sensitivity to detect four clinically relevant groups (HbAA, HbA/β-thalassemia, HbAS, and SCD) for the Gazelle, HemoTypeSC, Sickle SCAN and the automated sickling test were 97%, 74%, 75%, and 76%, respectively. Furthermore, the overall or balanced sensitivity for the automated sickling test was 85% to detect three groups relevant for screening—1) normal (HbAA), 2) trait (HbAS and HbA/β-thalassemia), and 3) disease (HbS/β-thalassemia and HbSS).

### Concordance between low-cost tests

Among all the low-cost tests, the lateral flow assays (HemoTypeSC and Sickle SCAN) had the same possible outcomes for phenotypes, *i.e.* HbAA, HbAS, HbSS, HbSC, and HbAC (although HbC was not considered in this study). The concordance between the results from HemoTypeSC and Sickle SCAN was 99%, with only one discordant result (HbAS sample detected as HbAS in Sickle SCAN and HbSS in HemoTypeSC). Similarly, the concordance between the solubility test and the conventional sickling test, both of which only detected the presence of HbS, was 99% with one erroneous case for the conventional sickling test where RBC sickling (and presence of HbS) was not detected in an SCT sample.

### Comparison of detection techniques

**Fig. 2:**
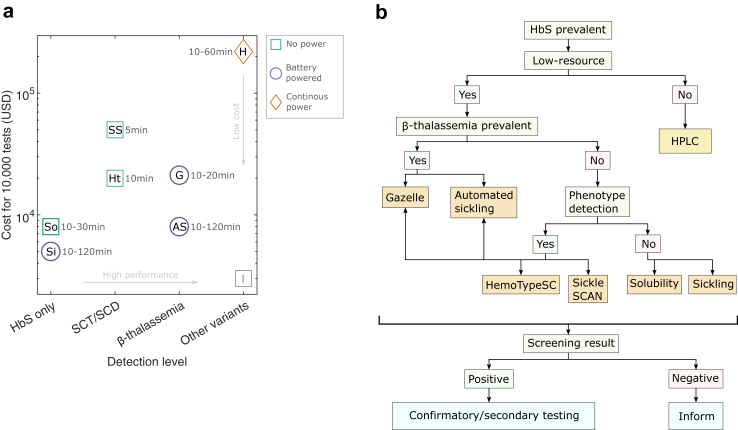
Comparison of different techniques and recommendations. a) Cost versus performance plot for 6 low-cost tests and the reference test (Hb HPLC), with shapes indicating power requirement and time required for testing shown next to different tests. The cost estimates for screening 10,000 individuals is based on expenses of running the study in Nepal and Canada, which includes costs of goods and consumables, and cost for one piece of equipment (for sickling tests, Gazelle and HPLC), and excludes costs related to transport, training, infrastructure, etc. The fees for technicians and technologists running the tests are also not included in the estimates, except for estimates for HPLC, which are based on the quoted costs for running HPLC in the hospital/clinic that account for operator fees. I, Ideal; Si, Sickling; So, Solubility; AS, Automated sickling; Ht, HemotypeSC; SS, Sickle SCAN; G, Gazelle; H, High performance liquid chromatography. b) Recommended guide to selecting low-cost test for screening sickle cell disease and β-thalassemia in rural/remote and low-resource settings.

Each detection technique described here has its strengths and shortcomings. In terms of detection capability, the conventional sickling test and the solubility test can detect the presence of HbS, but not of β-thalassemia or HbA; the lateral flow assays (HemoTypeSC, Sickle SCAN) can detect phenotypes for normal, SCT and SCD (but not β-thalassemia); the Gazelle and the automated sickling test can detect phenotypes for normal, SCD due to HbSS and HbS/β-thalassemia, SCT, and β-thalassemia trait; while Hb HPLC can detect the previously mentioned phenotypes as well as other Hb variants that lead to SCD when coinherited with HbS, *e.g.* HbD^Punjab^ (Fig. 2a). In terms of material costs or other expenses during the study in Nepal and Canada (between 2022 and 2023), the solubility test and the sickling test (conventional and automated) costed less than US$1 per test (1 US$ = around 126 Nepalese Rupee in 2022), with an additional cost of US$1000–3000 for the microscope used in the sickling test. HemoTypeSC and Gazelle costed US$2 per test (with an additional US$1200 for the Gazelle portable reader), and Sickle SCAN costed US$5 per test. The costs for the low-cost techniques listed above and in Fig. 2a do not include expenses related to technologist time, transport, training, infrastructure, and additional consumables (*e.g.* vortex mixer for Gazelle; vials for HemoTypeSC, pipettes, pipette tips, *etc.*). The reference test, Hb HPLC costed up to US$10–30 per test (including costs for reagents, technologist time, and maintenance), and the Hb HPLC instrument at the respective clinical sites costed up to tens of thousands of dollars.

In terms of electrical power requirement, the lateral flow assays and the solubility test did not require electrical power for operation, while equipment for the sickling test (conventional and automated) and the Gazelle were battery powered. The HPLC instrument required a dedicated laboratory and an electrical outlet for continuous power. In terms of time, the lateral flow assays took 5–10 min to display bands for phenotypes and the solubility test took 10–30 min; and these tests also allowed for preparation of multiple samples simultaneously while awaiting test results. For the sickling test, the prepared slides were imaged 2 h after preparation for observing sickled vs. round red blood cells (conventional) or for morphology-based classification (automated). With sequential sample preparation, after the initial wait time (120 min), slides for participants were imaged every 2–5 min. The Gazelle took 10–20 min per test and did not allow for simultaneous testing or sample preparation. For Hb HPLC testing, although the BioRad Variant II could hold up to five sample racks (50 samples in total) at a time, sample racks accommodating up to 10 samples were loaded individually onto either of the Hb HPLC systems in Nepal or Canada and tested within 1 h without the need for technologist supervision. For field implementation, there could be additional time required (possibly up to a few hours or even days) for confirmatory HPLC tests if individuals are screened in remote or rural locations without access to HPLC equipment, and such a delay is not required for the low-cost tests, which are portable.

## Discussion

In rural or remote and low-resource settings, where the burden of SCD and β-thalassemia is high, accurate low-cost tests to detect carrier and disease conditions are needed to break the diagnostic barrier. In regions with high prevalence of HbS but low prevalence of β-thalassemia, any of the low-cost tests evaluated here can be used as a screening tool, with Gazelle and lateral flow assays having the highest sensitivity (>96%) for detecting phenotypes HbAA, HbAS and HbSS (Fig. 2b). Thus, factors such as budget, availability of trained personnel, time, ease of use, and regulatory approvals can be considered to select the optimal technique.

In regions with high prevalence of both HbS and β-thalassemia, however, the optimal low-cost test should be selected with caution, particularly to avoid false negative results for β-thalassemia trait, which may result in compound heterozygous SCD (*i.e.* HbS/β-thalassemia) in the next generation. Out of all the low-cost tests, only the Gazelle and the automated sickling test detected β-thalassemia trait (Fig. 2), with sensitivity of 91% and 72%, respectively. Furthermore, using a different machine learning model or classifier, the automated sickling test identified trait conditions (HbAS and HbA/β-thalassemia) grouped together with an increased sensitivity of 85%. Aside from the Gazelle and the automated sickling test, the other low-cost tests are only suitable for areas with high HbS and β-thalassemia prevalence in conjunction with other low-cost tests intended to detect β-thalassemia, such as NESTROFT.^26^ Note that unlike sensitivity and specificity, the positive and negative predictive values (PPV and NPV) reported in Tables 2 and 3 depend on the prevalence of the disease or condition in the population being tested. As the prevalence of a condition (*e.g.*, HbS or β-thalassemia) increases, the PPV to detect that condition increases while the NPV decreases.^27^

All the low-cost tests that identified phenotypes (Gazelle, HemoTypeSC, Sickle SCAN and automated sickling test) were accurate at detecting severe disease cases, including HbSS and HbS/β-thalassemia, with sensitivity and specificity of over 96% and 99%, respectively. Hence, any of these four tests can serve as a potential screening tool in low-resource settings for quick identification of patients with severe SCD that need access to treatment or support, including government-based initiatives. For instance, in Nepal, although the government has allocated Nepalese Rupee NRs. 100,000 (∼ USD $719 in 2025) to patients with SCD for treatment and medical support,^28^ many individuals in rural or remote regions still remain undiagnosed or misdiagnosed; thus implementing accurate, low-cost screening options (such as the four tests) can help increase access to such support.

The results for the automated sickling test in Table 3 are presented for fixed thresholds of the classifiers considered. However, for machine-learning based classification, several variations could be chosen, *e.g.* for a different set of groups (HbAA + HbA/β-thalassemia, HbAS, and SCD), the overall sensitivity and specificity increased to 89% and 95%, respectively. Additionally, the classifier threshold can be modified (*e.g.* resulting in higher sensitivity and lower specificity for disease or carrier forms where confirmatory tests are available) based on the implementation needs.^23^

There were a few limitations of the study. First, although the suggested low-cost tests are intended to be implemented in the field, our studies in Nepal and Canada were conducted in well-equipped clinical laboratory settings, since this research study required access to Hb HPLC as the reference test. However, all the low-cost tests evaluated here are portable and therefore a feasibility study outlining the benefits and practical challenges of implementing these low-cost tests in resource-constrained settings will be valuable. Second, our analysis did not include β-thalassemia major patients, and our recruitment did not include other haemoglobin variants not prevalent in Nepal (*e.g.* HbC). Additionally, variants such as HbE, which are present in Nepal,^29^^,^^30^ were not considered in this study even though compound heterozygous forms such as HbE/β-thalassemia can have severe clinical manifestations. Moreover, it is likely that the morphology of RBCs for HbE trait or HbEE is like that of RBCs for β-thalassemia trait. Finally, our study did not recruit newborns and children under two years of age. It is imperative however to include newborn screening in disease diagnosis and management programs, and the feasibility of newborn screening with these low-cost tests should be evaluated, as previously done for a few of the techniques discussed here.^10^^,^^12^

Overall, the findings of the present study can help inform researchers and health practitioners on potential alternatives to expensive and inaccessible gold standard tests for screening sickle cell disease and β-thalassemia in low-resource settings.

## Contributors

Pranav Shrestha: Conceptualisation, Methodology, Software, Formal analysis, Investigation, Writing–Original Draft, Writing–Review & Editing, Visualisation, Funding acquisition; Hendrik Lohse: Software, Investigation, Writing–Review & Editing; Christopher Bhatla: Conceptualisation, Methodology, Investigation, Writing–Review & Editing. Heather McCartney: Resources, Project administration; Alaa Alzaki: Resources. Navdeep Sandhu: Resources, Project administration. Pradip Kumar Oli: Investigation, Project administration; Sanjeev Chaudhary: Investigation, Project administration; Ali Amid: Methodology, Resources, Writing–Review & Editing; Rodrigo Onell and Nicholas Au: Conceptualisation, Methodology, Writing–Review & Editing; Hayley Merkeley: Conceptualisation, Methodology, Resources, Writing–Review & Editing; Videsh Kapoor: Conceptualisation, Methodology, Writing–Review & Editing, Funding acquisition; Rajan Pande: Conceptualisation, Methodology, Resources, Supervision; Boris Stoeber: Conceptualisation, Methodology, Formal analysis, Writing–Original Draft, Writing–Review & Editing, Supervision, Funding acquisition.

## Data sharing statement

The data that support the findings of this study are available in the Supplementary Information of this article. The raw or processed data for the automated microscopy and machine-learning based detection is available at https://doi.org/10.20383/103.0916.^31^

## Declaration of interests

This study was supported by the UBC Public Scholars Initiative (PSI) (provided to BS). This research was undertaken, in part, with support from UBC Health Innovation Funding Investment (HIFI) Awards (provided to BS), and the UBC Four Year Doctoral Fellowship (4YF) program (stipend to PS). BS held a Canada Research Chair (CRC) position that provided partial teaching release (giving more time for research, thereby indirectly supporting this project). The UBC Centre for Blood Research is home to the Naiman Vickars Endowment fund which has provided funds for this project and partly funds Dr. Ali Amid's research. RO is a Member of Royal College examining board and Member of Diagnostic Accreditation Program in British Columbia. We declare no other competing interests.

## Appendix A Supplementary data

Supplementary

Translated abstract Nepali
