# Supplementary material for: Evaluation of low-cost techniques to detect sickle cell disease and β-thalassemia: an open-label, international, multicentre study
Source: Lancet Reg Health Southeast Asia. 2025 Mar 29;35:100571. doi: 10.1016/j.lansea.2025.100571 (PMC11994944; doi:10.1016/j.lansea.2025.100571)
Supplement: Supplementary [file mmc1.docx]

Evaluation of low-cost techniques to detect sickle cell disease and β-thalassemia: an open-label, international, multicentre study

*Pranav Shrestha, Hendrik Lohse, Christopher Bhatla, Heather McCartney, Alaa Alzaki, Navdeep Sandhu, Pardip Kumar Oli, Sanjeev Chaudhary, Ali Amid, Rodrigo Onell, Nicholas Au, Hayley Merkeley, Videsh Kapoor, Rajan Pande, Boris Stoeber*

Contents

[1. Blood sample collection 1](#_Toc191846260)

[2. Testing procedure for low-cost tests 2](#_Toc191846261)

[2.1 HbS Solubility Test 2](#_Toc191846262)

[2.2 HemoTypeSC 2](#_Toc191846263)

[2.3 Sickle SCAN 2](#_Toc191846264)

[2.4 Gazelle Hb Variant Test 2](#_Toc191846265)

[2.5 Conventional and automated sickling test 2](#_Toc191846266)

[3. Data analysis details 3](#_Toc191846267)

[3.1 Calculation of 95% confidence intervals (95% CI) 4](#_Toc191846268)

[4. HPLC results summary 4](#_Toc191846269)

[5. Analysis by hemoglobin variant 5](#_Toc191846270)

[6. Analysis by phenotype – 5 groups 8](#_Toc191846271)

[7. Analysis by phenotype – 4 (or 3) groups 9](#_Toc191846272)

[8. References 12](#_Toc191846273)

# 1. Blood sample collection

Trained phlebotomists or healthcare technologists collected samples of whole blood, which were stored in collection tubes with anticoagulant ethylenediaminetetraacetic acid (EDTA) – 4.0 mL vacuum blood collection tubes with K2 EDTA (BD Vacutainer, Becton, Dickinson and Company) in Canada, and 3.0 mL disposable non-vacuum blood collection tubes with K3 EDTA (AV LabotUbe, AV Consumables) in Nepal. De-identified blood samples were stored at a temperature of 4°C, and analyses were performed within 1-7 days of blood collection. Prior to analysis, the samples were placed at room temperature (20-25°C) for at least 30 minutes, and gently mixed by inverting the tubes or placing them in rotary mixers.

# 2. Testing procedure for low-cost tests

## 2.1 HbS Solubility Test

The deoxygenated form of HbS is insoluble in phosphate buffer and causes the solution (blood mixed with reagent) to be turbid, indicating a positive test result, for SCT or SCD cases. In the absence of HbS, the solution is clear (negative test result)(1). The testing procedure included mixing 20 µL of whole blood with 2 mL of the reagent in a tube or vial, and waiting for 10 minutes to check the solution turbidity against a marked sheet/card with black horizontal lines on a white background. For a positive result, the black lines were not visible through the turbid solution, while for a negative result, the black lines were visible through the clear solution.

## 2.2 HemoTypeSC

Six drops of distilled water were added to a test vial, using the provided dropper pipette. A drop of blood (20 µL) was placed on a glass slide, and the white pad of the blood sampling device was placed on the drop to absorb it into the sample pad. The blood sampling device was placed into the test vial with distilled water, and the vial was swirled to transfer blood components to the water, ensuring that the water turned pink or light-red in color. While the blood sampling device was in the test vial, the HemoTypeSC test strip was inserted into the test vial. After 10 minutes, the test strip was removed to read the results.

## 2.3 Sickle SCAN

As per the instructions for use, the testing procedure included mixing blood with a provided buffer solution to lyse red blood cells, adding 5 drops of the treated sample to the inlet of the Sickle SCAN cartridge and reading the results after around 5 minutes.

## 2.4 Gazelle Hb Variant Test

Firstly, 20 µL of whole blood was mixed with 40 µL of marker fluid in an Eppendorf tube using a vortex mixer for 20 seconds. Then 50 µL of buffer was added to wet the testing cartridge paper, while holding the cartridge at a 35-45° angle from the horizontal and placing it on a stand for 60 seconds. A stamper soaked the blood and marker fluid mixture, and was placed on the cartridge for 5 seconds. Two wells at the ends of the cartridge were then filled with 200 µL of buffer each, and the cartridge was placed on a portable reader, which performed the test and displayed the result in around 7-8 minutes. All the details of the testing procedure had to be carefully followed to avoid errors and incomplete tests, which mostly occurred as a result of improper wetting of the cartridge paper.

## 2.5 Conventional and automated sickling test

Whole blood (150 µL) was mixed with a sickling reagent (300 µL of 2% sodium metabisulphite) in a separate tube/vial. A volume of 3 µL to 3.3 µL of the mixture was pipetted onto a microscope slide, and a coverslip was gently placed to spread the mixture evenly. The wet preparation was sealed by spreading a mixture of melted VALAP (Vaseline, Lanolin and Parrafin in 1:1:1 mass ratio)(2), and the process was repeated to prepare a total of two coverslips on a slide. Up to 225 images per coverslip were captured two hours after sample preparation using a low-cost automated microscope, Octopi (Open configurable high-throughput imaging platform for infectious disease diagnosis in the field, Prakash Lab, Stanford)(3). For each participant, 6 to 12 coverslips were imaged. The captured images were also evaluated by eye, referred to here as the conventional sickling test. For the automated sickling test, the cells in each image were segmented using a human-in-the-loop approach in Cellpose 2.0(4), and each coverslip of the participant was classified into different participant groups using morphology-based machine learning in MATLAB (MathWorks Inc., Massachusetts, USA).

Around 30 different machine learning models were trained and tested on coverslip level with 6-12 coverslips per patient. The learning process entails patient level splits, and to assess the repeatability and variability between different splits, the learning is repeated independently for 1000 iterations of randomized splits. This avoids reporting an overly positive result from a particular random split as the result can be split dependent. The values reported (e.g. for sensitivity, specificity, etc.) are the mean for 1000 iterations, which is more representative of the method and the entire dataset. In terms of variability between the 1000 iterations, the 95% CI range was typically < 1-2% for the variables reported (sensitivity, specificity, PPV and NPV, as defined in the following section). This variability is relatively low and ultimately could also justify the use of a random split, but the mean of the 1000 random splits is more representative of the entire dataset.

Within an iteration, the entire data set is split into training and testing datasets (80:20) at the patient level, such that coverslips for the same patient do not appear in both training and testing datasets for that iteration. Within the training dataset (which is part of the 80, in the 80:20 split), k-fold cross-validation (with k=10) is applied for each iteration. Here, the different folds are created randomly on all the coverslips within the training set in each iteration, so for cross-validation, the folds are not necessarily split at the patient level. For this reason, it is essential to perform the patient-level data split for the training and testing datasets (80:20 split).

An important consideration is that there is no data leakage (patient-level) between training and test sets, neither within an iteration nor across iterations. For each iteration, data from the test set remains completely unseen during training and validation for that iteration. Data from the same participants may end up in both training and testing sets across different iterations, but these iterations are independent of each other, and there is no data leakage or feedback between iterations. Without any feedback between iterations, the model does not indirectly learn from test data (causing potentially overly optimistic performance estimates).

# 3. Data analysis details

The test results are categorized according to the following definitions: i) true positive (TP) indicates a positive result for both the index test and the reference test; ii) true negative (TN) indicates a negative result for both the index test and the reference test; iii) false positive (FP) indicates a positive result for the index test, but a negative result for the reference test; iv) false negative (FN) indicates a negative result for the index test, but a positive result for the reference test. The reference test in this study was Hb HPLC. All the tests (including index tests and reference test) were done using unique participant identifiers, and the clinical information of the participants were not included in these identifiers.

The sensitivity = TP/(TP+FN)×100%, measures the ability of the index test to correctly identify participants with the condition. For screening efforts in communities with undiagnosed individuals, the sensitivity of the disease and trait conditions should be high, so that such individuals are not missed during screening. After screening, it is possible to identify false positive cases using follow-up confirmatory/secondary tests.

The specificity = TN/(FP+TN)×100%, measures the ability of the index test to correctly identify people without the condition.

The positive predictive value, PPV= TP/(TP+FP)×100%, is the probability that a positively screened individual actually has the condition, and the negative predictive value, NPV= TN/(TN+FN)×100%, is the probability that a negatively screened individual actually does not have the condition.

## 3.1 Calculation of 95% confidence intervals (95% CI)

Let’s denote the sample estimate for a proportion (sensitivity, specificity, PPV, or NPV) to be $p$, which is estimating the population proportion $\pi$. The estimate for proportion (calculated in the study), $p=r/n$, relates to the number of individuals with the particular characteristic $r$ and the sample size $n$. In this study, the following proportions are calculated(8):

| $\boldsymbol{p}$ | $\boldsymbol{r}$ | $\boldsymbol{n}$ |
| --- | --- | --- |
| Sensitivity | TP | TP + FN |
| Specificity | TN | TN + FP |
| PPV | TP | TP + FP |
| NPV | TN | TN + FN |

The Wilson confidence intervals (CI),

| $\frac{p+u/2}{u+1}\pm\frac{z_{crit}}{u+1}\sqrt{\frac{p(1-p)}{n}+\frac{u}{4n}}$ | (1) |
| --- | --- |

can be calculated using the estimate of the proportion $p$, the sample size $n$, $z_{crit}=1.96$ (for 95% CI), and $u=z_{crit}^{2}/n$.

# 4. HPLC results summary

The mean, standard deviation and range (minimum and maximum) for the major peaks of different hemoglobin variants (HbA, HbF, HbA_2_, HbS) from Hb HPLC results for all 138 participants in Nepal and Canada are listed in Table S1.

**Table S1** Summary of HPLC results (area % for peaks of HbA, HbF, HbA_2_, and HbS) for Nepal (N) and Canada (C) for the different phenotypes, HbAA (AA), HbA/β-thalassemia (A/β), HbAS (AS), HbS/β-thalassemia (S/β) and HbSS (SS). The table contains minimum (min), maximum (max), mean, standard deviation (st dev), median, and interquartile range (IQR).

|  |  |  | **HbA** (Area %) | | | | | **HbF** (Area %) | | | | |
| --- | --- | --- | --- | --- | --- | --- | --- | --- | --- | --- | --- | --- |
|  |  | n | min | max | Q1 | median | Q3 | min | max | Q1 | median | Q3 |
|  | AA | 24 | 83.40 | 86.70 | 84.30 | 84.85 | 85.33 | 0.00 | 1.00 | 0.00 | 1.00 | 0.85 |
|  | A/β | 23 | 79.70 | 83.60 | 80.70 | 81.00 | 81.60 | 0.00 | 3.70 | 0.88 | 3.70 | 1.53 |
| **N** | AS | 36 | 52.00 | 65.80 | 59.05 | 60.35 | 61.58 | 0.90 | 7.60 | 1.10 | 7.60 | 2.30 |
|  | S/β | 10 | 3.20 | 6.80 | 3.43 | 3.50 | 3.93 | 0.00 | 21.10 | 0.00 | 21.10 | 17.95 |
|  | SS | 18 | 2.20 | 8.30 | 3.13 | 3.60 | 4.00 | 0.00 | 20.90 | 10.35 | 20.90 | 19.15 |
|  | AA | 6 | 85.40 | 87.80 | 86.55 | 86.70 | 87.25 | 0.20 | 0.70 | 0.25 | 0.30 | 0.5 |
| **C** | AS | 9 | 50.70 | 62.50 | 51.3 | 52.80 | 57.1 | 0.10 | 1.90 | 0.3 | 0.40 | 0.4 |
|  | S/β | 1 | 1.30 | 1.30 |  | 1.30 |  | 30.20 | 30.20 |  | 30.20 |  |
|  | SS | 11 | 1.50 | 12.10 | 1.9 | 2.20 | 2.75 | 1.60 | 29.00 | 4.25 | 13.90 | 17.8 |

|  |  |  | **HbA_2_** (Area %) | | | | | **HbS** (Area %) | | | | |
| --- | --- | --- | --- | --- | --- | --- | --- | --- | --- | --- | --- | --- |
|  |  | n | min | max | Q1 | median | Q3 | min | max | Q1 | median | Q3 |
|  | AA | 24 | 2.30 | 3.10 | 2.68 | 2.95 | 3.00 | 0.00 | 0.00 | 0.00 | 0.00 | 0.00 |
|  | A/β | 23 | 4.90 | 7.20 | 5.70 | 6.00 | 6.45 | 0.00 | 0.00 | 0.00 | 0.00 | 0.00 |
| **N** | AS | 36 | 2.90 | 4.50 | 3.28 | 3.50 | 3.73 | 19.00 | 35.20 | 23.98 | 25.15 | 26.20 |
|  | S/β | 10 | 4.10 | 5.80 | 4.48 | 4.90 | 5.00 | 58.90 | 69.70 | 62.43 | 64.95 | 65.80 |
|  | SS | 18 | 1.90 | 3.80 | 2.40 | 2.90 | 3.20 | 47.60 | 79.10 | 63.43 | 70.65 | 75.83 |
|  | AA | 6 | 2.50 | 2.90 | 2.6 | 2.70 | 2.7 | 0.00 | 0.00 | 0.00 | 0.00 | 0.00 |
| **C** | AS | 9 | 2.50 | 3.50 | 2.8 | 3.10 | 3.3 | 26.40 | 41.40 | 33.6 | 38.40 | 38.8 |
|  | S/β | 1 | 4.20 | 4.20 |  | 4.20 |  | 64.30 | 64.30 |  | 64.30 |  |
|  | SS | 11 | 2.30 | 3.60 | 3.05 | 3.30 | 3.35 | 66.10 | 93.00 | 76.2 | 82.20 | 86.15 |

Note: A cut-off of around 4% for HbA_2_ was used as the local diagnostic criteria for β-thalassemia in Nepal. Cases with HbA_2_ between 3.6% and 4% were considered borderline, and recommended for molecular testing of genotype. Based on Table S1, HbA_2_ ≥ 4.9% for study population with HbA/β-thalassemia and HbA_2_ ≥ 4.1% for study population with HbS/β-thalassemia.

# 5. Analysis by hemoglobin variant

The values for true positive, true negative, false positive, false negative, sensitivity, specificity, positive predictive value (PPV), negative predictive value (NPV) are provided for Gazelle (Table S2), HemoTypeSC (Table S3), Sickle SCAN (Table S4), Solubility test (Table S5), and Conventional sickling test (Table S6). The following definitions apply:

- True positive (TP): Both reference test and index test contain Hb variant (can be heterozygous or homozygous)
- True negative (TN): Both reference test and index test do no contain Hb variant, regardless of other variants present
- False positive (FP): Reference test does not contain Hb variant, but index test contains Hb variant (heterozygous or homozygous)
- False negative (FN): Reference test contains Hb variant (heterozygous or homozygous), but index test does not contain Hb variant

**Table S2** Analysis by hemoglobin variant for Gazelle, *i.e.* detection of the presence of HbA in HbAA, HbA/β-thalassemia or HbAS; presence of HbS in HbS/β-thalassemia or HbSS; and presence of β in HbA/β-thalassemia or HbS/β-thalassemia

|  | A | S | β |  |  |
| --- | --- | --- | --- | --- | --- |
| **True Positive** | 98 | 85 | 21 |  |  |
| **True Negative** | 40 | 53 | 102 |  |  |
| **False Positive** | 0 | 0 | 2 |  |  |
| **False Negative** | 0 | 0 | 13 |  |  |
| **Total** | 138 | 138 | 138 | **Overall (A,S,β)** | **Overall (A,S)** |
| **Sensitivity (%)** | 100 (96-100) | 100 (96-100) | 62 (45-76) | **87 (78-93)** | **100 (96-100)** |
| **Specificity (%)** | 100 (91-100) | 100 (93-100) | 98 (93-99) | **99 (93-100)** | **100 (92-100)** |
| **PPV (%)** | 100 (96-100) | 100 (96-100) | 91 (73-98) | **97 (90-99)** | **100 (96-100)** |
| **NPV (%)** | 100 (91-100) | 100 (93-100) | 89 (82-93) | **96 (89-99)** | **100 (92-100)** |

**Table S3** Analysis by hemoglobin variant for HemoTypeSC, *i.e.* detection of the presence of HbA in HbAA, HbA/β-thalassemia or HbAS; presence of HbS in HbS/β-thalassemia or HbSS; and presence of β in HbA/β-thalassemia or HbS/β-thalassemia

|  | A | S | β |  |  |
| --- | --- | --- | --- | --- | --- |
| **True Positive** | 97 | 85 | 0 |  |  |
| **True Negative** | 40 | 53 | 104 |  |  |
| **False Positive** | 0 | 0 | 0 |  |  |
| **False Negative** | 1 | 0 | 34 |  |  |
| **Total** | 138 | 138 | 138 | **Overall (A,S,β)** | **Overall (A,S)** |
| **Sensitivity (%)** | 99 (94-100) | 100 (96-100) | 0 (0-10) | **66 (55-76)** | **100 (95-100)** |
| **Specificity (%)** | 100 (91-100) | 100 (93-100) | 100 (96-100) | **100 (94-100)** | **100 (92-100)** |
| **PPV (%)** | 100 (96-100) | 100 (96-100) | - | **-** | **100 (96-100)** |
| **NPV (%)** | 98 (87-100) | 100 (93-100) | 75 (68-82) | **91 (83-96)** | **99 (90-100)** |

**Table S4** Analysis by hemoglobin variant for Sickle SCAN, *i.e.* detection of the presence of HbA in HbAA, HbA/β-thalassemia or HbAS; presence of HbS in HbS/β-thalassemia or HbSS; and presence of β in HbA/β-thalassemia or HbS/β-thalassemia

|  | A | S | β |  |  |
| --- | --- | --- | --- | --- | --- |
| **True Positive** | 98 | 85 | 0 |  |  |
| **True Negative** | 40 | 53 | 104 |  |  |
| **False Positive** | 0 | 0 | 0 |  |  |
| **False Negative** | 0 | 0 | 34 |  |  |
| **Total** | 138 | 138 | 138 | **Overall (A,S,β)** | **Overall (A,S)** |
| **Sensitivity (%)** | 100 (96-100) | 100 (96-100) | 0 (0-10) | **67 (55-76)** | **100 (96-100)** |
| **Specificity (%)** | 100 (91-100) | 100 (93-100) | 100 (96-100) | **100 (94-100)** | **100 (92-100)** |
| **PPV (%)** | 100 (96-100) | 100 (96-100) | - | **-** | **100 (96-100)** |
| **NPV (%)** | 100 (91-100) | 100 (93-100) | 75 (68-82) | **92 (84-96)** | **100 (92-100)** |

**Table S5** Analysis by hemoglobin variant for solubility test, *i.e.* detection of the presence of HbA in HbAA, HbA/β-thalassemia or HbAS; presence of HbS in HbS/β-thalassemia or HbSS; and presence of β in HbA/β-thalassemia or HbS/β-thalassemia

|  | A | S | β |  |  |
| --- | --- | --- | --- | --- | --- |
| **True Positive** | 53 | 85 | 0 |  |  |
| **True Negative** | 40 | 53 | 104 |  |  |
| **False Positive** | 0 | 0 | 0 |  |  |
| **False Negative** | 45 | 0 | 34 |  |  |
| **Total** | 138 | 138 | 138 | **Overall (A,S,β)** | **Overall (A,S)** |
| **Sensitivity (%)** | 54 (44-64) | 100 (96-100) | 0 (0-10) | **51 (40-63)** | **77 (67-84)** |
| **Specificity (%)** | 100 (91-100) | 100 (93-100) | 100 (96-100) | **100 (94-100)** | **100 (92-100)** |
| **PPV (%)** | 100 (93-100) | 100 (96-100) | - | **-** | **100 (95-100)** |
| **NPV (%)** | 47 (37-58) | 100 (93-100) | 75 (68-82) | **74 (64-82)** | **74 (62-82)** |

**Table S6** Analysis by hemoglobin variant for conventional sickling test, *i.e.* detection of the presence of HbA in HbAA, HbA/β-thalassemia or HbAS; presence of HbS in HbS/β-thalassemia or HbSS; and presence of β in HbA/β-thalassemia or HbS/β-thalassemia

|  | A | S | β |  |  |
| --- | --- | --- | --- | --- | --- |
| **True Positive** | 54 | 84 | 0 |  |  |
| **True Negative** | 40 | 53 | 104 |  |  |
| **False Positive** | 0 | 0 | 0 |  |  |
| **False Negative** | 44 | 1 | 34 |  |  |
| **Total** | 138 | 138 | 138 | **Overall (A,S,β)** | **Overall (A,S)** |
| **Sensitivity (%)** | 55 (45-65) | 99 (94-100) | 0 (0-10) | **51 (40-62)** | **77 (67-84)** |
| **Specificity (%)** | 100 (91-100) | 100 (93-100) | 100 (96-100) | **100 (94-100)** | **100 (92-100)** |
| **PPV (%)** | 100 (93-100) | 100 (96-100) | - | **-** | **100 (95-100)** |
| **NPV (%)** | 48 (37-58) | 98 (90-100) | 75 (68-82) | **74 (64-82)** | **73 (61-82)** |

# 6. Analysis by phenotype – 5 groups

The confusion matrices and calculations for true positive, true negative, false positive, false negative, sensitivity, specificity, positive predictive value (PPV), negative predictive value (NPV) are provided for Gazelle (Table S7), HemoTypeSC (Table S8), and Sickle SCAN (Table S9).

**Table S7** Analysis by phenotype (5 groups) variant for Gazelle, *i.e.* detection of the presence of HbAA, HbAS, HbSS, HbS/β-thalassemia or HbA/β-thalassemia

|  | **Gazelle** (Index test) | | | | | |  |
| --- | --- | --- | --- | --- | --- | --- | --- |
| **HPLC**  (Reference test) |  | AA | AS | SS | Sβ | Aβ | **Total** |
|  | AA | 29 | 0 | 0 | 0 | 1 | 30 |
|  | AS | 0 | 45 | 0 | 0 | 0 | 45 |
|  | SS | 0 | 0 | 28 | 1 | 0 | 29 |
|  | Sβ | 0 | 0 | 11 | 0 | 0 | 11 |
|  | Aβ | 2 | 0 | 0 | 0 | 21 | 23 |
|  | Total | 31 | 45 | 39 | 1 | 22 | 138 |
|  | True Positive | 29 | 45 | 28 | 0 | 21 |  |
|  | True Negative | 106 | 93 | 98 | 126 | 114 |  |
|  | False Positive | 2 | 0 | 11 | 1 | 1 |  |
|  | False Negative | 1 | 0 | 1 | 11 | 2 | **Overall** |
|  | Sensitivity (%) | 97 (83-99) | 100 (92-100) | 97 (83-99) | 0 (0-26) | 91 (73-98) | **77 (59-89)** |
|  | Specificity (%) | 98 (93-99) | 100 (96-100) | 90 (83-94) | 99 (96-100) | 99 (95-100) | **97 (92-99)** |
|  | PPV (%) | 94 (79-98) | 100 (92-100) | 72 (56-83) | 0 (0-79) | 96 (78-99) | **72 (54-85)** |
|  | NPV (%) | 99 (95-100) | 100 (96-100) | 99 (95-100) | 92 (86-95) | 98 (94-100) | **98 (93-99)** |

**Table S8** Analysis by phenotype (5 groups) variant for HemoTypeSC, *i.e.* detection of the presence of HbAA, HbAS, HbSS, HbS/β-thalassemia or HbA/β-thalassemia

|  | **HemoTypeSC** (Index test) | | | | | |  |
| --- | --- | --- | --- | --- | --- | --- | --- |
| **HPLC**  (Reference test) |  | AA | AS | SS | Sβ | Aβ | Total |
|  | AA | 30 | 0 | 0 | 0 | 0 | 30 |
|  | AS | 0 | 44 | 1 | 0 | 0 | 45 |
|  | SS | 0 | 0 | 29 | 0 | 0 | 29 |
|  | Sβ | 0 | 0 | 11 | 0 | 0 | 11 |
|  | Aβ | 23 | 0 | 0 | 0 | 0 | 23 |
|  | Total | 53 | 44 | 41 | 0 | 0 | 138 |
|  | True Positive | 30 | 44 | 29 | 0 | 0 |  |
|  | True Negative | 85 | 93 | 97 | 127 | 115 |  |
|  | False Positive | 23 | 0 | 12 | 0 | 0 |  |
|  | False Negative | 0 | 1 | 0 | 11 | 23 | **Overall** |
|  | Sensitivity (%) | 100 (89-100) | 98 (88-100) | 100 (88-100) | 0 (0-26) | 0 (0-14) | **60 (41-76)** |
|  | Specificity (%) | 79 (70-85) | 100 (96-100) | 89 (82-94) | 100 (97-100) | 100 (97-100) | **94 (87-97)** |
|  | PPV (%) | 57 (43-69) | 100 (92-100) | 71 (55-82) | - | - | **-** |
|  | NPV (%) | 100 (96-100) | 99 (94-100) | 100 (96-100) | 92 (86-95) | 83 (76-89) | **95 (89-98)** |

**Table S9** Analysis by phenotype (5 groups) variant for Sickle SCAN, *i.e.* detection of the presence of HbAA, HbAS, HbSS, HbS/β-thalassemia or HbA/β-thalassemia

|  | **Sickle SCAN** (Index test) | | | | | |  |
| --- | --- | --- | --- | --- | --- | --- | --- |
| **HPLC**  (Reference test) |  | AA | AS | SS | Sβ | Aβ | Total |
|  | AA | 30 | 0 | 0 | 0 | 0 | 30 |
|  | AS | 0 | 45 | 0 | 0 | 0 | 45 |
|  | SS | 0 | 0 | 29 | 0 | 0 | 29 |
|  | Sβ | 0 | 0 | 11 | 0 | 0 | 11 |
|  | Aβ | 23 | 0 | 0 | 0 | 0 | 23 |
|  | Total | 53 | 45 | 40 | 0 | 0 | 138 |
|  | True Positive | 30 | 45 | 29 | 0 | 0 |  |
|  | True Negative | 85 | 93 | 98 | 127 | 115 |  |
|  | False Positive | 23 | 0 | 11 | 0 | 0 |  |
|  | False Negative | 0 | 0 | 0 | 11 | 23 | **Overall** |
|  | Sensitivity (%) | 100 (89-100) | 100 (92-100) | 100 (88-100) | 0 (0-26) | 0 (0-14) | **60 (42-76)** |
|  | Specificity (%) | 79 (70-85) | 100 (96-100) | 90 (83-94) | 100 (97-100) | 100 (97-100) | **94 (88-97)** |
|  | PPV (%) | 57 (43-69) | 100 (92-100) | 73 (57-84) | - | - | **-** |
|  | NPV (%) | 100 (96-100) | 100 (96-100) | 100 (96-100) | 92 (86-95) | 83 (76-89) | **95 (89-98)** |

# 7. Analysis by phenotype – 4 (or 3) groups

The confusion matrices and calculations for true positive, true negative, false positive, false negative, sensitivity, specificity, positive predictive value (PPV), negative predictive value (NPV) are provided for Gazelle (Table S10), HemoTypeSC (Table S11), Sickle SCAN (Table S12), and Automated sickling test using 4 groups (Table S13) and 3 groups (Table S14).

**Table S10** Analysis by phenotype (4 groups) variant for Gazelle, *i.e.* detection of the presence of HbAA, HbAS, SCD (HbSS, HbS/β-thalassemia) or HbA/β-thalassemia

|  | **Gazelle** (Index test) | | | | |  |
| --- | --- | --- | --- | --- | --- | --- |
| **HPLC**  (Reference test) |  | AA | AS | SCD | Aβ | **Total** |
|  | AA | 29 | 0 | 0 | 1 | 30 |
|  | AS | 0 | 45 | 0 | 0 | 45 |
|  | SCD | 0 | 0 | 40 | 0 | 40 |
|  | Aβ | 2 | 0 | 0 | 21 | 23 |
|  | Total | 31 | 45 | 40 | 22 | 138 |
|  | True Positive | 29 | 45 | 40 | 21 |  |
|  | True Negative | 106 | 93 | 98 | 114 |  |
|  | False Positive | 2 | 0 | 0 | 1 |  |
|  | False Negative | 1 | 0 | 0 | 2 | **Overall** |
|  | Sensitivity (%) | 97 (83-99) | 100 (92-100) | 100 (91-100) | 91 (73-98) | **97 (85-99)** |
|  | Specificity (%) | 98 (93-99) | 100 (96-100) | 100 (96-100) | 99 (95-100) | **99 (95-100)** |
|  | PPV (%) | 94 (79-98) | 100 (92-100) | 100 (91-100) | 96 (78-99) | **97 (86-100)** |
|  | NPV (%) | 99 (95-100) | 100 (96-100) | 100 (96-100) | 98 (94-100) | **99 (95-100)** |

**Table S11** Analysis by phenotype (4 groups) variant for HemoTypeSC, *i.e.* detection of the presence of HbAA, HbAS, SCD (HbSS, HbS/β-thalassemia) or HbA/β-thalassemia

|  | **HemoTypeSC** (Index test) | | | | |  |
| --- | --- | --- | --- | --- | --- | --- |
| **HPLC**  (Reference test) |  | AA | AS | SCD | Aβ | Total |
|  | AA | 30 | 0 | 0 | 0 | 30 |
|  | AS | 0 | 44 | 1 | 0 | 45 |
|  | SCD | 0 | 0 | 40 | 0 | 40 |
|  | Aβ | 23 | 0 | 0 | 0 | 23 |
|  | Total | 53 | 44 | 41 | 0 | 138 |
|  | True Positive | 30 | 44 | 40 | 0 |  |
|  | True Negative | 85 | 93 | 97 | 115 |  |
|  | False Positive | 23 | 0 | 1 | 0 |  |
|  | False Negative | 0 | 1 | 0 | 23 | **Overall** |
|  | Sensitivity (%) | 100 (89-100) | 98 (88-100) | 100 (91-100) | 0 (0-14) | **74 (58-86)** |
|  | Specificity (%) | 79 (70-85) | 100 (96-100) | 99 (94-100) | 100 (97-100) | **94 (88-97)** |
|  | PPV (%) | 57 (43-69) | 100 (92-100) | 98 (87-100) | - | **-** |
|  | NPV (%) | 100 (96-100) | 99 (94-100) | 100 (96-100) | 83 (76-89) | **96 (90-98)** |

**Table S12** Analysis by phenotype (4 groups) variant for Sickle SCAN, *i.e.* detection of the presence of HbAA, HbAS, SCD (HbSS, HbS/β-thalassemia) or HbA/β-thalassemia

|  | **Sickle SCAN** (Index test) | | | | |  |
| --- | --- | --- | --- | --- | --- | --- |
| **HPLC**  (Reference test) |  | AA | AS | SCD | Aβ | Total |
|  | AA | 30 | 0 | 0 | 0 | 30 |
|  | AS | 0 | 45 | 0 | 0 | 45 |
|  | SCD | 0 | 0 | 40 | 0 | 40 |
|  | Aβ | 23 | 0 | 0 | 0 | 23 |
|  | Total | 53 | 45 | 40 | 0 | 138 |
|  | True Positive | 30 | 45 | 40 | 0 |  |
|  | True Negative | 85 | 93 | 98 | 115 |  |
|  | False Positive | 23 | 0 | 0 | 0 |  |
|  | False Negative | 0 | 0 | 0 | 23 | **Overall** |
|  | Sensitivity (%) | 100 (89-100) | 100 (92-100) | 100 (91-100) | 0 (0-14) | **75 (59-86)** |
|  | Specificity (%) | 79 (70-85) | 100 (96-100) | 100 (96-100) | 100 (97-100) | **95 (89-98)** |
|  | PPV (%) | 57 (43-69) | 100 (92-100) | 100 (91-100) | - | **-** |
|  | NPV (%) | 100 (96-100) | 100 (96-100) | 100 (96-100) | 83 (76-89) | **96 (90-98)** |

**Table S13** Analysis by phenotype (4 groups) for automated sickling test, i.e. detection of the presence of HbAA, HbAS, SCD (HbSS, HbS/β-thalassemia) or HbA/β-thalassemia

|  | **Automated sickling** (Index test) | | | | |  |
| --- | --- | --- | --- | --- | --- | --- |
| **HPLC**  (Reference test) |  | AA | Aβ | AS | SCD | Total |
|  | AA | 42800 | 18912 | 7238 | 59 | 69009 |
|  | Aβ | 10124 | 49408 | 9477 | 0 | 69009 |
|  | AS | 7374 | 8533 | 52024 | 1078 | 69009 |
|  | SCD | 677 | 177 | 1369 | 66786 | 69009 |
|  | Total | 60975 | 77030 | 70108 | 67923 | 276036 |
|  | True Positive | 42800 | 49408 | 52024 | 66786 |  |
|  | True Negative | 188852 | 179405 | 188943 | 205890 |  |
|  | False Positive | 18175 | 27622 | 18084 | 1137 |  |
|  | False Negative | 26209 | 19601 | 16985 | 2223 | **Overall** |
|  | Sensitivity (%) | 62 (50-73) | 72 (60-81) | 75 (64-84) | 97 (90-99) | **76 (65-85)** |
|  | Specificity (%) | 91 (87-94) | 87 (81-91) | 91 (87-94) | 100 (97-100) | **92 (88-95)** |
|  | PPV (%) | 70 (58-80) | 64 (53-74) | 74 (63-83) | 98 (92-100) | **77 (65-85)** |
|  | NPV (%) | 88 (83-92) | 90 (85-94) | 92 (87-95) | 99 (96-100) | **92 (88-95)** |

**Table S14** Analysis by phenotype (3 groups) for automated sickling test, i.e. detection of the presence of HbAA, trait (HbAS, HbA/β-thalassemia), or SCD (HbSS, HbS/β-thalassemia)

|  | **Automated sickling test** (Index test) | | | |  |
| --- | --- | --- | --- | --- | --- |
| **HPLC**  (Reference test) |  | AA | Trait | SCD | Total |
|  | AA | 69177 | 27260 | 286 | 96723 |
|  | Trait | 12972 | 82109 | 1642 | 96723 |
|  | SCD | 276 | 2338 | 94115 | 96729 |
|  | Total | 82425 | 111707 | 96043 | 290175 |
|  | True Positive | 69177 | 82109 | 94115 |  |
|  | True Negative | 180204 | 163854 | 191518 |  |
|  | False Positive | 13248 | 29598 | 1928 |  |
|  | False Negative | 27546 | 14614 | 2614 | **Overall** |
|  | Sensitivity (%) | 72 (62-80) | 85 (76-91) | 97 (92-99) | **85 (76-90)** |
|  | Specificity (%) | 93 (89-96) | 85 (79-89) | 99 (96-100) | **92 (88-95)** |
|  | PPV (%) | 84 (74-90) | 74 (65-81) | 98 (93-99) | **85 (77-91)** |
|  | NPV (%) | 87 (81-91) | 92 (87-95) | 99 (96-100) | **92 (88-95)** |

Note: Machine learning based classification can be modified based on type of classifier, combinations of groups tested, level of data aggregation for morphological characterization (e.g. image-level, coverslip-level, slide-level, donor-level), misclassification penalties (that allow training the model to avoid specific types of misclassifications, *e.g.* misclassifying disease or trait as normal), etc.

# 8. References

1. Wild BJ, Bain BJ. Investigation of Variant Haemoglobins and Thalassaemias. In: Dacie and Lewis Practical Haematology [Internet]. Twelfth Ed. Elsevier; 2017. p. 282–311. Available from: http://dx.doi.org/10.1016/B978-0-7020-6696-2.00014-X

2. Valap Sealant. Cold Spring Harb Protoc [Internet]. 2015 Feb 2;2015(2):pdb.rec082917. Available from: http://www.cshprotocols.org/lookup/doi/10.1101/pdb.rec082917

3. Li H, Soto-Montoya H, Voisin M, Valenzuela LF, Prakash M. Octopi: Open configurable high-throughput imaging platform for infectious disease diagnosis in the field. bioRxiv [Internet]. 2019; Available from: https://doi.org/10.1101/684423

4. Pachitariu M, Stringer C. Cellpose 2.0: how to train your own model. Nat Methods [Internet]. 2022 Nov 7;(April):2022.04.01.486764. Available from: https://www.biorxiv.org/content/10.1101/2022.04.01.486764v1%0Ahttps://www.biorxiv.org/content/10.1101/2022.04.01.486764v1%0Ahttps://www.biorxiv.org/content/10.1101/2022.04.01.486764v1.abstract

5. Kuhn M, Johnson K. Applied Predictive Modeling [Internet]. New York, NY: Springer New York; 2013. Available from: http://link.springer.com/10.1007/978-1-4614-6849-3

6. Shrestha P, Lohse H, Bhatla C, Onell R, Au NHC, Amid A, et al. Low-Cost Automated Microscopy and Morphology-Based Machine Learning Classification of Sickle Cell Disease and Beta-Thalassemia in Nepal and Canada. Blood [Internet]. 2023 Nov 28;142(Supplement 1):790–790. Available from: http://dx.doi.org/10.1182/blood-2023-187754

7. Shrestha P, Lohse H, Bhatla C, Mccartney H, Alzaki A, Oli PK, et al. Morphology-based classification of sickle cell disease and β- thalassemia using a low-cost automated microscope and machine learning. medRxiv. 2024;1–32.

8. Walters SJ, Campbell MJ, Machin D. Medical Statistics: A Textbook for the Health Sciences. Fifth edit. Wiley Blackwell; 2021.
