# Supplementary material for: Evaluation of low-cost techniques to detect sickle cell disease and β-thalassemia: an open-label, international, multicentre study
Source: Lancet Reg Health Southeast Asia. 2025 Mar 29;35:100571. doi: 10.1016/j.lansea.2025.100571 (PMC11994944; doi:10.1016/j.lansea.2025.100571)
Supplement: Translated abstract Nepali [file mmc2.docx]

This translation in Nepali language was submitted by the authors and we reproduce it as supplied. It has not been peer reviewed. Our editorial processes have only been applied to the original abstract in English, which should serve as reference for this manuscript.

**सिकलसेल रोग र β-थ्यालेसेमिया पत्ता लगाउने कम लागतका प्राविधिक उपायहरुको मूल्याङ्कन: एक अन्तराष्ट्रिय अध्ययन बहु-केन्द्र**

# सारांस

**पृष्टभुमि**: सिकलसेल रोग एउटा प्रमुख विश्वब्यपि स्वास्थ्य समस्या रहेको छ जसले कम र मध्यम आय गर्ने मुलुकका केटाकेटीहरुलाई अप्रत्यासित अनुपातमा असर गरेको छ। यस्ता मुलुकहरुको लागि हेमोग्लोबिन एस (HbS) तथा β‍-थ्यालेमेसिया जस्ता अन्य जीवाणुका बाहक वा रोगका प्रकारहरु पत्ता लताउनको लागि स्थानीय उपचारकेन्द्रहरुमा तत्काल उपलब्ध गराउन सकिने कम लागतका प्राविधिक उपायहरुको शिघ्र आवश्यक छ।

**विधी**: हेमोग्लोबिनोपाथिज (HbA/β-thalassemia, HbAS, HbS/β-thalassemia, HbSS) भएको जानकारीमा आइसकेका स्वस्थ स्वयंसेवक र सहभागीहरुबाट रगत नमुनाहरु संकलन गरिएको थियो। छ वटा कम लागतका परीक्षणहरुले देखाएका नतिजालाई अन्य स्थापित प्रविधिहरुबाट उपलब्ध नतीजाहरु (HPLC; ClinicalTrials.gov Identifier: NCT05506358) सँग तुलाना गरेर मुल्यांकन गरिएको थियो।

**निष्कर्शहरु:** सेप्टेम्बर २०२२ देखि मार्च २०२३ सम्ममा हामीले २ देखि ७४ वर्ष उमेर समुहका (५९% महिला ४१% पुरुष) १३८ सहभागीहरुलाई यस अध्ययनको लागि नेपाल र क्यानाडाका उपचार स्थलहरुमा सूचिक्रित गरेका थियौं। चार प्रकारका कम लागतका परीक्षणहरु (HemoTypeSC, Sickle SCAN, Gazelle, and automated sickling) जसले फेनोटाइपहरुलाई पहिचान गर्न सकेको र सिकलसेल रोगको (HbSS, HbS/β-thalassemia) सहि तरिकाले (sensitivity >96%; specificity >99%) पत्ता लगाएको थियो। यसको तुलनामा HemotypeSC र Sickle SCAN ले HbAS (sensitivity >97%; specificity 100%) मात्र पत्ता लगाएको तर HbA/β-thalassemia (sensitivity 0%; specificity 100%) लाई पत्ता लगाउन सकेको थिएन तर Gazelle ले भने HbAS (sensitivity 100%, specificity 100%) and HbA/β-thalassemia (sensitivity 91%, specificity 99%) र स्वचालित सिकलिंग परीक्षणले दुबै प्रकारको अवस्थाहरु (HbAS and HbA/β-thalassemia; sensitivity 85%, specificity 85%) पत्ता लगाएको थियो।

**तर्क तथा व‍िश्लेषण**: जब सिकलसेल र β-थ्यालेसेमिया संयुक्त उपस्थिति हुन्छ गेजेल्ले र स्वचालित सिकलिंगको (Gazelle and automated sickling) परीक्षणले नाजुक सिकलसेल रोग र त्यसको बाहकलाई विश्वसनीय तवरले पत्ता लगाउँछ। तर HemotypeSC र Sickle SCAN ले β-थ्यालेसेमियाको उपस्थिति पत्ता लगाउन नसक्ने भएकोले अन्य कम लागतका परीक्षणहरु समेट्न आवश्यक हुन्छ।

**आर्थिक सहयोग**: ब्रिटिश कोलम्बिया विश्वविद्यालय PSI, क्यानडा अनुसन्धान अध्यक्षहरु, ब्रिटिश कोलम्बिया विश्वविद्यालय HIFI Award, ब्रिटिश कोलम्बिया विश्वविद्यालय 4YF, Naiman Vickars Endowment fund ।
